# Nobiletin suppresses cell viability through AKT Pathways in PC-3 and DU-145 prostate cancer cells

**DOI:** 10.1186/2050-6511-15-59

**Published:** 2014-10-24

**Authors:** Jianchu Chen, Ashley Creed, Allen Y Chen, Haizhi Huang, Zhaoliang Li, Gary O Rankin, Xingqian Ye, Guihua Xu, Yi Charlie Chen

**Affiliations:** 1 College of Biosystems Engineering and Food Science, Fuli Institute of Food Science, Zhejiang Key Laboratory for Agro-Food Processing, Zhejiang University, Hangzhou 310058, China; 2 College of Science, Technology and Mathematics, Alderson Broaddus University, Philippi, WV 26416, USA; 3 Department of Pharmaceutical Science, West Virginia University, Morgantown, WV 26506, USA; 4 Department of Pharmacology, Physiology and Toxicology, Joan C. Edwards School of Medicine, Marshall University, Huntington, WV 25755, USA

**Keywords:** Nobiletin, Prostate cancer, VEGF, NF-κB, HIF-1α, cMyc

## Abstract

**Background:**

Nobiletin is a non-toxic dietary flavonoid that possesses anti-cancer properties. Nobiletin has been reported to reduce the risk of prostate cancer, but the mechanism is not well understood. In this study, we investigated the effects of nobiletin in prostate cancer cell lines PC-3 and DU-145.

**Methods:**

Nobiletin was isolated from a polymethoxy flavonoid mixture using HPLC, cell viability was analyzed with MTS-based assays. Protein expression was examined by ELISA and western blotting. Gene expression was examined by luciferase assay. And the pathways were examined by manipulating genetic components with plasmid transfection.

**Results:**

Data showed that nobiletin decreased cell viability in both prostate cell lines, with a greater reduction in viability in PC-3 cells. HIF-1α expression and AKT phosphorylation were decreased in both cell lines. The VEGF expression was inhibited in PC-3 but not DU-145 cells. cMyc expression was decreased in DU-145 cells. Nobiletin down-regulated NF-κB (p50) expression in nuclei of DU145 cells but not whole cells. It also suppressed NF-κB expression in both whole cells and nuclei of PC-3 cells. Increasing HIF-1α levels reversed nobiletin’s inhibitory effects on VEGF expression, and up-regulating AKT levels reversed its inhibitory effects on HIF-1α expression. We speculate that AKT influences cell viability probably by its effect on NF-κB in both prostate cells. The effect of nobiletin on VEGF expression in PC-3 cell lines was through the AKT/HIF-1α pathway.

**Conclusion:**

Taken together, our results show that nobiletin suppresses cell viability through AKT pathways, with a more profound effect against the more metastatic PC-3 line. Due to this enhanced action against a more malignant cell type, nobiletin may be used to improve prostate cancer survival rates.

## Background

Prostate cancer is the second most common cancer in the United States, as well as, the second leading cause of mortality among males in the western world [1]. Approximately 25% of all newly diagnosed cancers in American men are prostate cancer [2]. Studies relating lifestyle to the risk of prostate cancer have become more prevalent in recent years due the escalating number of prostate cancer cases over the past decade [1]. Nevertheless, the etiology of prostate cancer is still uncertain because no specific carcinogen is known to cause this disease [3]. Research has found that certain risk factors, such as advancing age, African American ethnicity, and a positive family history, are associated with the likelihood of developing prostate cancer [4]. However, research has also shown that prostate cancer is not solely due to genetic factors, but is also related to lifestyle, diet, and environmental factors [4-6]. It is now believed that 90-95% of all cancers are caused by lifestyle [7]. This observation has encouraged researchers to identify dietary components, such as flavonoids like nobiletin, which may have anticancer properties.

It has been suggested that dietary intake of natural products rich in citrus flavonoids can play an important role in chemoprevention [8,9]. Flavonoids are phytochemicals found in fruits, vegetables, teas, and wines. Flavonoids display anti-carcinogenic characteristics *in vitro* and might be able to decrease cancer risk by changing levels of sex hormones, preventing oxidation or inflammation, diminishing angiogenesis or cell proliferation, or stimulating apoptosis [10]. There are more than 400 flavonoids found in our food supply; however, in this research we focused our attention on nobiletin [11].

Nobiletin is an O-methylated flavonoid found in citrus peels with an empirical formula of C_21_H_22_O_8_ and molecular weight of 402.39 [12]. An inverse relationship has been identified between nobiletin and cancer risk, which is likely due to nobiletin’s anticancer, antiviral, and anti-inflammatory activities [13,14]. More specifically, recent findings have identified nobiletin as a cell differentiation modulator. Cell differentiation is a crucial step in angiogenesis and therefore could affect tumor growth and metastasis which both depend on angiogenesis [15]. Research has also shown that a diet high in flavonoids reduced oxidative damage to deoxyribonucleic acid (DNA), blocking a significant step in the onset of some types of cancers [16]. These findings support the proposition that nobiletin is functionally unique and could be a possible chemopreventive agent in inflammation-associated tumorigenesis [17].

Currently, metastatic prostate cancer is incurable and ultimately claims the life of patients [18,19]. An important factor in the relative seriousness of prostate cancer is the invasiveness of the constituent tumor cells causing metastasis [19]. Nobiletin has been reported to reduce the risk of prostate cancer, but the mechanism is not well understood. Therefore we studied the effects of nobiletin in prostate cancer cell lines PC-3 and DU-145. The pathways that affect the viability and VEGF expression of these cell lines have also been investigated in this paper. DU-145 and PC-3 are prostate cancer cell lines with moderate and high metastatic potential, respectively [20]. In the present study, we isolated nobiletin from a polymethoxy flavonoid mixture. Then we investigated the effect of nobiletin on cell viability in prostate cancer cell lines PC-3 and DU-145 and also performed western blotting and ELISA to identify changes in protein expression. Moreover, we examined the VEGF changes through transfection of AKT and HIF-1α plasmids in luciferase assays.

## Methods

### Cell culture and treatment

PC-3 cells were cultured in F-12K medium (ATCC, Manassas, VA) supplemented with 10% US-qualified fetal bovine serum (FBS) (Invitrogen, Grand Island, NY). DU-145 cells were cultured in Eagle’s minimum essential medium (ATCC, Manassas, VA) supplemented with 10% US-qualified fetal bovine serum. All cells were cultured in a cell culture incubator with 5% CO_2_ at 37°C. Nobiletin was dissolved in dimethyl sulfoxide (DMSO) to make stock solutions of 100 mM and equal amount of DMSO was included in controls for every experiment.

### Cell proliferation assay

Effects of nobiletin on prostate cancer cells (PC-3 and DU-145) viability were colorimetrically determined with a “Cell Titer 96 Aqueous One Solution Cell Proliferation Assay” kit from Promega (Madison, WI). Cells (5 × 10^3^/well) were seeded into 96-well plates and incubated for 16 h before being treated with 0 to 160 μg/ml nobiletin in triplicates for 24 h with DMSO as solvent control. After removing the medium, cells were washed with phosphate buffered saline (PBS), and then 100μL Aqueous One Reagent dilute solution (80 μL PBS +20 μL Aqueous One Reagent) was added to each well. Cells were incubated at 37°C for 1.5 h and measured for optical density (OD) values at 490 nm. Cell viability was expressed as a percentage of control from three independent experiments.

### ELISA for VEGF

Secreted vascular endothelial growth factor (VEGF) protein levels were analyzed by sandwich enzyme-linked immunosorbent assay (ELISA) with a Quantikine Human VEGF Immunoassay Kit from R&D Systems (Minneapolis, MN) targeting VEGF in cell culture supernates. Cells (10^4^/well) were seeded into 96-well plates and incubated for 16 h before being treated with 0 to 160 μg/ml nobiletin in triplicates for 24 h with DMSO as solvent control. Culture supernates were collected for VEGF assay. VEGF levels were determined following the manufacturer's instructions. A total of 3 independent experiments, each in triplicates, were assayed, and the mean VEGF protein level from each duplicate was used for statistical analysis.

### Western blot

Prostate cancer cells (10^6^) were seeded in 60-mm dishes and incubated for 16 h before treatment with nobiletin for 24 h. After washing with PBS, cells were harvested with 100 μL Mammalian Protein Extraction Reagent including 1 μL Halt Protease, 1 μL Phosphatase Inhibitor and 2 μL EDTA (Thermo Scientific, Rockford, IL). Cells were then frozen at -80°C for 30 min, melted, centrifuged at 12,000 g at 4°C for 10 min, and collected in aqueous phase for measurement. Nuclear protein was extracted by NE-PER™ Nuclear and Cytoplasmic Extraction Reagents (Thermo Scientific, Rockford, IL). Total protein levels were assayed with a BCA Protein Assay Kit (Pierce, Rockford, IL), and lysates were separated by 10% SDS-PAGE and blotted into nitrocellulose membrane. For immune detection, antibodies against HIF-1α, NF-κB (p50), PTEN, cMyc, GAPDH, p-AKT, total AKT (Santa Cruz Biotechnology, Santa Cruz, CA) and PCNA (Cell Signaling Technology, Boston, MA) were applied and signals visualized with x-ray film (Pierce Biotechnology, Rockford, IL). Protein bands were quantitated with NIH ImageJ software, normalized to corresponding GAPDH, PCNA or total AKT bands, and expressed as percentages of control. A total of three independent experiments were carried out for statistical analysis.

### Transient transfection and luciferase assay

PC-3 prostate cancer cells were seeded in 96-well plate at 10^4^ cells/well and incubated overnight. The cells were then transfected with 0.05 μg VEGF (Hif-1α) luciferase reporter, 0-0.25 μg HIF-1α (AKT) or SR-α plasmids by 0.6 μL jetPRIME reagent (VWR, West Chester, PA) for 4 hours, followed by 16 hour treatment with 0 or 40 μM nobiletin. The cells were harvested and analyzed for luciferase and total protein levels, and the levels of VEGF (HIF-1α) reporter were normalized by corresponding total protein levels. Data represent Means ± SE from three independent experiments.

### Statistical analysis

Results were expressed as mean ± standard error of mean (SEM). Statistical assessment was carried out with the program system of SPSS (Version 16.0 for Windows). The results were analyzed using one-way analysis of variance (ANOVA) and post hoc test (2-sided Dunnett's t) to test both overall differences and specific differences between each treatment and control. A *p* value of less than 0.05 was considered significant.

## Results

### Isolation and identification of nobiletin

**Figure 1:**
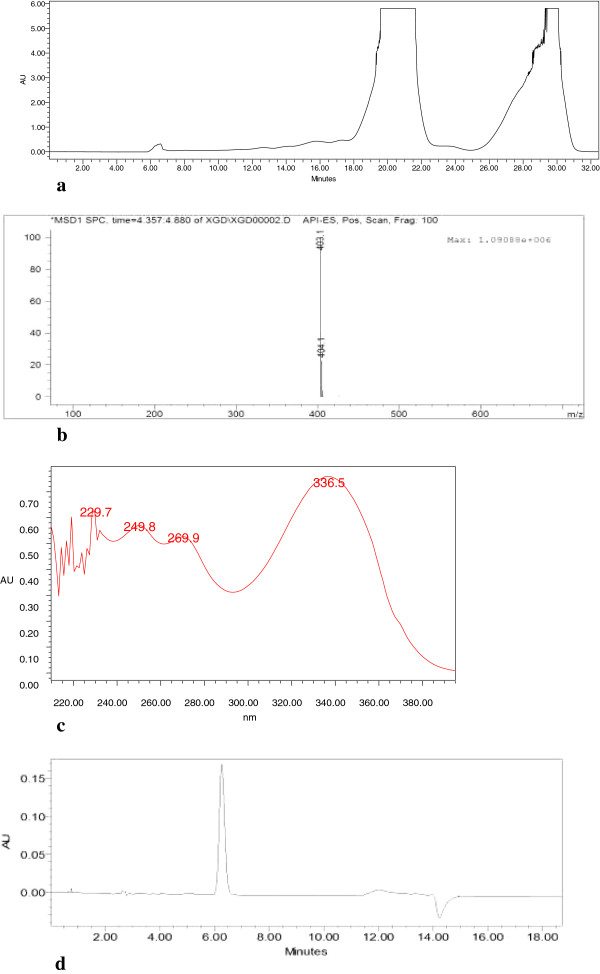
**Isolation and identification of nobiletin. (a)** Preparation HPLC graph of nobiletin and tangeretin. **(b)** HPLC-MS graph of nobiletin. **(c)** UV-vis chromatography of nobiletin. **(d)** HPLC graph of nobiletin.

Nobiletin was prepared from a polymethoxy flavonoid mixture, which was provided by Zhejiang Quzhou Tiansheng Plant Extraction Co. Ltd. in China, containing about 60% nobiletin and tangeretin. The polymethoxy flavonoid mixture was dissolved in methanol-dimethyl sulfoxide (1:1) to a concentration of 50 mg/mL. Then it was chromate graphed with high-performance liquid chromatography (HPLC), eluted with methanol-H_2_O (70:30) in 8 mL/min at room temperature, separated into two fractions (Fractions I and II), collected individually, and evaporated.Fraction I and fraction II were obtained by HPLC (Figure 1(a)). Fraction I was identified as nobiletin by HPLC-MS (Figure 1(b)), UV-vis chromatography (Figure 1(c)) and comparing peak time with that of nobiletin sample from Sigma (Figure 1(d)) and previous reports. Its purity was above 98%.

### Nobiletin inhibits cell viability in prostate cancer cell lines

**Figure 2:**
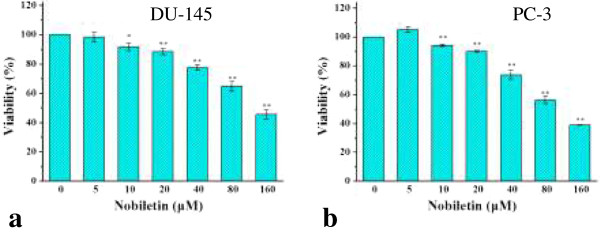
**Effect of nobiletin on viability of PC-3 and DU-145 cells. (a)** DU-145 cells, **(b)** PC-3 cells. Cells (5.0 × 10^3^ /well) were seeded in 96-well plates, incubated for 16 h, and treated with nobiletin for 24 h. Cell viability was colorimetrically determined by a MTS-based method and expressed as percentages of control. Data represents mean ± SE from 3 independent experiments. *P < 0.05 as compared to control. **P < 0.01 as compared to control.

Cell viability steadily decreased as nobiletin concentration increased in both cell lines (Figure 2). Beginning at a concentration of 10 μM nobiletin, PC-3 cell viability consistently decreased from 95% to 40% at a concentration of 160 μM nobiletin (p < 0.01). Similarly, DU-145 cell viability was also inhibited with each successive doubling of concentration. At a concentration of 10 μM nobiletin cell viability was 92% (p < 0.05), which was gradually inhibited to 46% by a 160 μM nobiletin treatment (p < 0.01). An overall inhibitory effect on cell viability was observed for both cell lines, although DU-145 (IC-50 = 137 μM nobiletin) cells appear more resistant than PC-3 cells (IC-50 = 117 μM nobiletin) to the inhibiting effect of nobiletin. It is in agreement with the cell viability determined by WST-1 assay [21].

### Nobiletin inhibits VEGF expression in prostate cancer cell line PC-3

**Figure 3:**
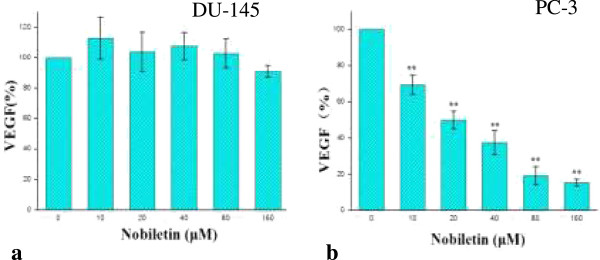
**Effect of nobiletin on VEGF expression in PC-3 and DU-145 cells. (a)** DU-145. **(b)** PC-3. Cells (1.0 × 10^4^/well) were seeded into 96-well plates , incubated for 16 h, and treated with nobiletin for 24 h. Vascular endothelial growth factor (VEGF) in cellculture supernate were analyzed with a Quantikine Human VEGF Immunoassay Kit from R&D Systems (Minneapolis, MN). Data represents mean ± SE from 3 independent experiments. *P < 0.05 as compared to control. **P < 0.01 as compared to control.

The levels of VEGF protein in PC-3 cell culture supernates were down-regulated to 70% at a concentration of 10 μM nobiletin (p < 0.01) and to 18% at a concentration of 160 μM nobiletin (p < 0.01) (Figure 3). However, the levels of VEGF protein in DU-145 cells ranged from 90-110% with no consistency with respect to nobiletin concentration. Our study revealed that VEGF expression was significantly (p < 0.01) reduced in PC-3 cancer cells by nobiletin treatment.

### Nobiletin inhibits HIF-1α (Hypoxia inducible factor) protein expression in prostate cancer cell lines

**Figure 4:**
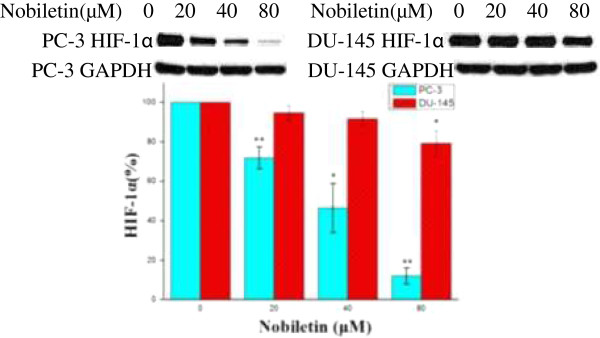
**Nobiletin's effects on HIF-1α expression in PC-3 and DU-145 cells.** Nobiletin's effect on HIF-1α expression. PC-3 and DU-145 cells (1.0 × 10^6^) were seeded in 60-mm dishes, incubated overnight, and treated with nobiletin for 24 hours. Cells were harvested and analyzed by SDS-PAGE and Western Blotting. HIF-1α protein levels were normalized by GAPDH protein levels. Data represents means ± SE from 3 independent experiments. *P < 0.05 compared to control. **P < 0.01 compared to control.

HIF-1α protein levels in PC-3 cells showed intense and consistent down-regulation by nobiletin treatment (Figure 4). A 20 μM nobiletin treatment led to inhibition of HIF-1α protein to 70% (p < 0.01). Higher concentrations of nobiletin resulted in greater inhibition, with the levels of HIF-1α protein down to 48% by a 40 μM nobiletin treatment (p < 0.05) and 10% by a 80 μM nobiletin treatment (p < 0.01). HIF-1α protein levels in DU-145 cells showed consistent but gradual down-regulation by nobiletin treatment. DU-145 cells seem to be much more resistant to nobiletin treatment with down-regulation ranging from 94% at a concentration of 20 μM nobiletin to 79% at a concentration of 80 μM nobiletin (p < 0.05). HIF-1α expression in both cells lines (PC-3 and DU-145) was inhibited by nobiletin treatment, with a greater inhibition in PC-3 cells.

### Nobiletin inhibits phosphorylation of AKT in prostate cancer cell lines

**Figure 5:**
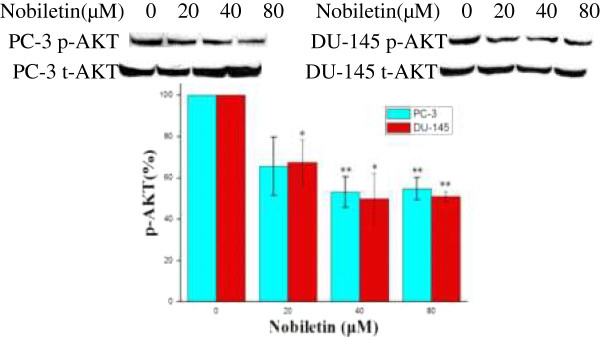
**Nobiletin's effect on AKT phosphorylation in PC-3 and DU-145 cells.** PC-3 and DU-145 cells (1.0 × 10^6^) were seeded in 60-mm dishes, incubated overnight, and treated with nobiletin for 24 hours. Cells were harvested and analyzed by SDS-PAGE and Western Blotting. P-AKT protein levels were normalized by total AKT protein levels and expressed as percentages of control. Data represents means ± SE from 3 independent experiments. *P < 0.05 compared to control. **P < 0.01 compared to control.

P-AKT levels were down-regulated from 65% by a 20 μM nobiletin treatment to 56% by a 80 μM nobiletin treatment (p < 0.01) in PC-3 cells (Figure 5). In DU-145 cells, p-AKT levels were down-regulated to 67% by a 20 μM nobiletin treatment (p < 0.05), to 51% by a 80 μM nobiletin treatment (p < 0.01). The percentage of down-regulation in each cell line was similar at each treatment concentration and indicates that nobiletin could decrease AKT phosphorylation for both PC-3 and DU-145. However, the observed down-regulation seemed to subside at 40 μM nobiletin treatment, with not much difference resulting at 80 μM nobiletin treatment for both cell lines. Therefore, higher concentrations may not be any more beneficial.

### Nobiletin inhibits cMyc expression in prostate cancer cell line DU-145

**Figure 6:**
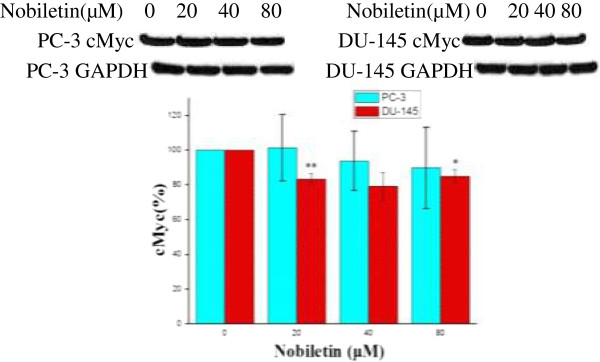
**Nobiletin’s effect on cMyc.** PC-3 and DU-145 cells (1.0 × 10^6^) were seeded in 60-mm dishes, incubated overnight, and treated with nobiletin for 24 hours. Cells were harvested and analyzed by SDS-PAGE and Western Blotting. cMyc levels were normalized by GAPDH protein levels and expressed as percentages of control. Data represents means ± SE from 3 independent experiments. *P < 0.05 compared to control. **P < 0.01 compared to control.

In PC-3 cells, cMyc levels were slightly down regulated to 93% by a 40 μM nobiletin treatment and to 90% by a 80 μM nobiletin treatment (Figure 6). The results were very gradual, revealing that the PC-3 cells have a high level of resistance and there was no statistical significance among treatments. The cMyc levels in DU-145 cells were down-regulated to 79-84% by nobiletin treatment. Therefore, nobiletin reduced cMyc expression in DU-145 cells, but showed no evidence of inhibition in PC-3 cells.

### Nobiletin inhibits NF-κB *(p50)* expression in nucleus of prostate cancer cell

**Figure 7:**
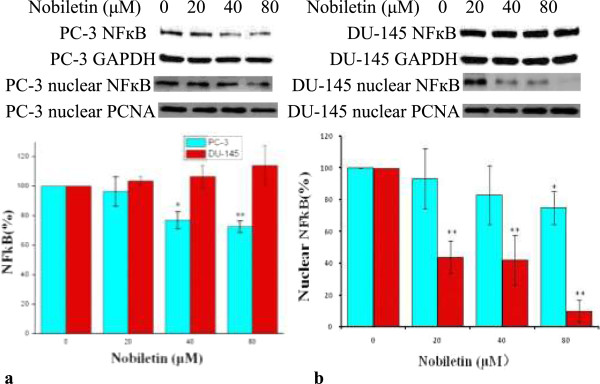
**Nobiletin's effect on NF-κB (p50) expression. (a)** Effect of nobiletin on total NF-kB expression, **(b)** Effect of nobiletin on nuclear NF-kB expression. PC-3 and DU-145 cells (1.0 × 10^6^) were seeded in 60 mm dishes, incubated overnight, and treated with nobiletin for 24 hours. Cells were harvested and analyzed by SDS-PAGE and Western Blotting. NF-кB protein levels whole cells were normalized by GAPDH protein levels and its protein levels of nucleus were normalized by PCNA protein levels. They were expressed as percentages of control. Data represents means ± SE from 3 independent experiments. *P < 0.05 compared to control. **P < 0.01 compared to control.

NF-κb (p50) expression was down-regulated in both whole cells and nuclei of PC-3 cells when treated with nobiletin. At the concentration of 80 μM nobiletin, its expression was inhibited by 28% (p < 0.01) and 37% (p < 0.05) respectively in whole cells and nuclei. In nuclei of DU145 cells, p50 expression was inhibited to 42% (p < 0.01) by a 40 μM nobiletin treatment and 10% (p < 0.01) by a 80 μM nobiletin treatment (Figure 7). However, its expression in whole cells was up-regulated to 103-112% with higher concentrations of nobiletin resulting in greater promotion.

### Effect of nobiletin on PTEN expression

**Figure 8:**
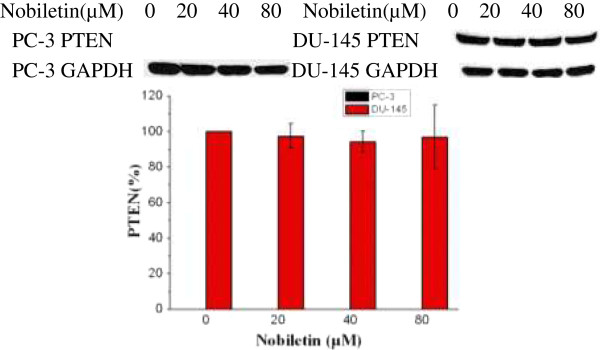
**Nobiletin’s effect on PTEN expression.** PC-3 and DU-145 cells (1.0 × 10^6^) were seeded in 60-mm dishes, incubated overnight, and treated with nobiletin for 24 hours. Cells were harvested and analyzed by SDS-PAGE and Western Blotting. PTEN protein levels were normalized by GAPDH protein levels and expressed as percentages of control. Data represents means ± SE from 3 independent experiments. *P < 0.05 compared to control. **P < 0.01 compared to control.

Our study found that nobiletin had no significant effect on PTEN expression in DU-145 cells (Figure 8). PTEN was not expressed in PC-3 cells.

### Nobiletin inhibits VEGF expression through regulating AKT and HIF-1α gene in prostate cancer cell line PC-3

**Figure 9:**
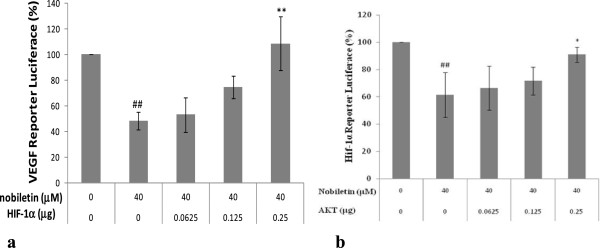
**Nobiletin inhibits VEGF expression by regulating AKT and HIF-1α gene in PC-3 cells. (a)** Nobiletin inhibits VEGF expression by regulating HIF-1 α gene, **(b)** Nobiletin inhibits VEGF expression by regulating AKT gene. PC-3 prostate cancer cells were seeded in 96-well plate at 10,000 cells/well and incubated overnight. The cells were then transfected with 0.05 ug VEGF (Hif-1α) luciferase reporter, 0-0.25 μg HIF-1α (AKT) or SR-α plasmids by 0.6 μL jetPRIME reagent for 4 hours, followed by 16 hour treatment with 0 or 40 μM nobiletin. The cells were harvested and analyzed for luciferase and total protein levels, and the levels of VEGF (Hif-1α) reporter were normalized by corresponding total protein levels. Data represent Means ± SE from 3 independent experiments. #p < 0.05 as compared to control. ##p <0.01 as compared to control. *p <0.05 as compared to nobiletin-treated control. **p <0.01 as compared to nobiletin-treated control.

We used PC-3 cancer cells with low levels of AKT and HIF-1α expression to test whether nobiletin affects VEGF expression. Our study found that transfected plasmid AKT and HIF-1α concentration-dependently reversed nobiletin’s inhibitory effects (Figure 9). Our findings suggest that nobiletin regulates VEGF expression through down-regulating AKT and HIF-1α in prostate cancer cells.

## Discussion

Approximately 25% of all newly diagnosed cancers in American men are prostate cancer [2]. The risk of developing prostate cancer is associated with advancing age, African American ethnicity, and a positive family history [4]. However, research has also shown that diet and other lifestyle factors may influence prostate cancer risk [4]. Studies relating lifestyle to the risk of prostate cancer have become more prevalent in recent years due to the escalating number of prostate cancer cases.

It has been suggested that dietary intake of natural products rich in flavonoids from citrus fruits may play a role in the prevention of cancer [8]. Tangeretin, nobiletin, hesperetin, hesperidin, naringenin, and naringin are just a few examples of citrus flavonoids that have the potential to be used as chemotherapeutic agents. Research has shown that these flavonoids possess inhibition activity on certain cancer cells' growth through various mechanisms [8].

Nobiletin, a citrus polymethoxy flavonoid, possesses anticancer, antiviral, and anti-inflammatory activities [14]. More specifically, recent findings have identified nobiletin as a cell differentiation modulator. Cell differentiation is a crucial step in angiogenesis and therefore could affect tumor growth and metastasis which both depend on angiogenesis [15]. These findings support the proposition that nobiletin is functionally unique and could be a possible chemopreventive agent in inflammation-associated tumorigenesis [17].

We tested the effectiveness of the preventive and/or treatment measures that nobiletin exhibits on PC-3 and DU-145 prostate cancer cells and showed that PC-3 and DU-145 cell viability was suppressed concentration-dependently by nobiletin treatment. Several pathways including VEGF, HIF-1α, AKT phosphorylation, cMyc, and NF-κB influence cell viability inhibition. In both cell lines, nobiletin inhibited phosphorylation of AKT, which is known to be the major signal for cell survival and proliferation [22]. Nobiletin treatment also reduced NF-κB (p50) expression in nuclei of both prostate cancer cells. NF-κB activation plays many roles when it enter into the nucleus, including initiating cellular transformation, mediating cellular proliferation, mediating cellular invasion and angiogenesis, mediating metastasis, and linking inflammation and cancer [23,24]. Suppression of NF-κB in tumor samples also inhibits proliferation, causes cell cycle arrest, and leads to apoptosis, indicating the crucial role of NF-κB in cell proliferation and survival [25]. Some researchers have found that the expression, activation and translocation of NF-κB were regulated by AKT pathways [26-28]. Our results showed that nobiletin treatment could decrease NF-κB expression in nuclei of both cells and AKT phosphorylation, indicating that AKT may influence cell viability by its effect on NF-κB in both prostate cells. It was also found that HIF-1α promoter is responsive to selective NF-κB subunits, indicating that NF-κB is a direct modulator of HIF-1α expression [29]. VEGF is a signal protein produced by cells related to vasculogenesis and angiogenesis, and it is also the downstream gene of HIF-1α. Our study revealed that VEGF expression was significantly (p < 0.01) reduced in PC-3 cancer cells by nobiletin treatment. HIF-1α expression in both cells lines (PC-3 and DU-145) was also inhibited by nobiletin treatment, with a greater inhibition in PC-3 cells. Furthermore, it was found that nobiletin inhibited VEGF expression through regulating AKT/HIF-1α pathways in prostate cancer cell line PC-3. Increasing HIF-1α levels actually reversed nobiletin’s inhibitory effects on VEGF expression. Similarly up-regulating AKT levels reversed its inhibitory effects on HIF-1α expression. These results correspond to a previous study that HIF-1α/VEGF expression can be regulated by AKT pathways [30]. Nobiletin reduced cMyc expression in DU-145 cells, but showed no evidence of inhibition in PC-3 cells. PTEN has been shown to play a pivotal role in apoptosis, cell cycle arrest, and possibly cell migration. PTEN is the most frequently mutated gene in prostate cancer, loss of heterozygosity at 10q23 can be detected in approximately 50% of human prostate cancers, whereas homozygous deletions of PTEN can be detected in approximately 10% of these cases [31]. However, nobiletin appears to lower cell viability through a mechanism independent of PTEN, as it does not seem to affect PTEN concentrations.

Our research indicated that nobiletin is a good candidate for the chemoprevention of prostate cancer in humans and could be an effective measure in inhibiting prostate cancer cell viability. Nobiletin has the apparent ability to suppress cell viability through multiple pathways, thus inhibiting tumor growth. Most encouraging is its capacity to suppress the more metastatic PC-3 cell line. Since the lethality of a tumor links directly to its ability to spread, nobiletin promises to increase the prostate cancer survival rate. However, more data needs to be obtained on nobiletin’s toxicity and tolerable dosages before it can become part of prostate cancer prevention and/or treatment. Also, as an in vitro model, cell culture cannot take absorption, distribution, metabolism, and excretion of nobiletin into consideration. Further studies in animal models and human trials are warranted to determine if physicians can promote this natural compound toward chemoprevention of prostate cancer cells.

## Conclusion

Our research indicated that nobiletin is a good candidate for the chemoprevention of prostate cancer in humans and could be an effective measure in suppressing prostate cancer cell viability. For these two prostate cancer cell lines, nobiletin has the apparent ability to suppress cell viability concentration-dependently through multiple pathways (VEGF, HIF-1α, AKT phosphorylation, cMyc, and NF-κB). Because nobiletin seems to work better against the more dangerous PC-3 cell line, nobiletin holds real potential in improving prostate cancer outcomes. However, more data needs to be obtained on nobiletin’s toxicity and tolerable dosages before it can become part of prostate cancer prevention and/or treatment. Also, as an in vitro model, cell culture cannot take absorption, distribution, metabolism, and excretion of nobiletin into consideration. Further studies in animal models and human trials are warranted to determine if physicians can promote this natural compound toward chemoprevention of prostate cancer cells.

## Competing interest

The authors state that they have no competing interest.

## Authors’ contributions

JC carried out the majority of experimental work. YC drafted the manuscript. All authors participated in experimental design and read and approved the final manuscript.

## Pre-publication history

The pre-publication history for this paper can be accessed here:

http://www.biomedcentral.com/2050-6511/15/59/prepub
